# Update on the Diagnosis and Treatment of Acute Appendicitis

**DOI:** 10.3390/jcm13237343

**Published:** 2024-12-02

**Authors:** George Vaos, Nikolaos Zavras

**Affiliations:** Medical School, National and Kapodistrian University of Athens, 10679 Athens, Greece; nzavras@med.uoa.gr

## 1. Introduction

Acute appendicitis (AA) is one of the most common surgical emergencies in adults and children [1]. The pathogenesis of AA is not clearly understood. The most accepted theory is that of intraluminal obstruction from various reasons that can lead to an increase in mucus production and bacterial overgrowth, resulting in wall tension, necrosis of the appendix, and perforation [1]. The diagnosis of AA is based on a synthesis of medical history, physical examination, laboratory evaluation, and imaging investigation [2]. Progressive pain, vomiting, nausea, lack of appetite, fever, guarding, and migration of the pain from the peri-umbilicus region in the lower right quadrant suggest AA [1,3]. However, the diagnosis of AA in children is challenging because of the atypical presentation and the difficulty of taking a trustworthy history and physical examination [4,5]. Similarly, in the adult population, the proportion of missed diagnoses of AA ranges between 20 and 40%, while the reported incidence of negative appendectomies ranges between 10 and 34% [6]. Currently, there is strong evidence that there are two types of AA: the uncomplicated AA (UAA) and the complicated AA (CAA) [7].

This Special Issue of the *Journal of Clinical Medicine* includes seven papers covering several topics across the AA spectrum: from perioperative outcomes in obese children undergoing appendectomy for UAA and CAA to a deep learning analysis of CT scan images to improve the detection of AA.

## 2. Outcomes of UAA and CAA in Children: Focus on Obesity

Obesity in childhood has become one of the most serious conditions in the 21st century. Obese children tend to be obese in adulthood, and they are at high risk for cardiac problems and diabetes mellites [8]. On the other hand, obesity may affect the patient’s response to surgical stress [9]. Ninh et al. [10] reported that obesity is a risk factor for sepsis and increased morbidity and mortality in adult patients after appendectomy.

Zavras et al. (contributor 1) conducted a systematic review investigating the impact of obesity on perioperative outcomes of obese children with UAA and CAA compared with normal-weight children. The authors did not find significant differences between the two groups apart from prolonged operative time, length of hospital stay, and surgical site infection in some articles [9,11,12]. 

## 3. Diagnosis of AA: Focus on Predicting Biomarkers

A substantial number of biomarkers, such as white blood cells (WBC), C-reactive protein (CRP), procalcitonin, calprotectin, the APPY1 biomarker panel (a combination of WBC, CRP, and myeloid reactive protein), red cell distribution width, pentraxin-3, intereleukin-6 (IL-6), the neutrophil-to-lymphocyte ratio, serum bilirubin, and the serum sodium levels, with different sensitivity and specificity rates, have been investigated for the diagnosis of AA [1,13,14,15,16]. In the current issue, in a systematic review and meta-analysis, Singh et al. (contributor 4) investigated the utility of serum ischemia-modified albumin (IMA) factor as a potential marker in the diagnosis of AA, differentiating it from other causes of acute abdominal pain, and as a marker to distinguish UAA from CAA. The authors showed that IMA is an applicable prognostic and diagnostic marker of AA.

## 4. Acute Appendicitis: Focus on the Controversies and Future Directions in the Management of AA

Although AA is a common lesion and has been investigated in a plethora of articles, several controversies in the diagnosis and treatment of AA still remain [17]. For example, issues that remain unresolved include the decision for operation or conservative treatment, indications for interval appendectomy, need for postoperative antibiotics, early versus delayed appendectomy for appendiceal phlegmon or abscess, and lack of protocols in the management of AA in neutropenic patients [17,18]. Dahiya et al. (contributor 3), discussed the current strategies and controversies in the management of AA in both adults and children. They pointed out a number of existing gaps in the current knowledge, such as the non-operative management of uncomplicated appendicitis, the role of interval appendectomy, the impact of delayed appendectomy on index admission, the role of preoperative and postoperative use of antibiotics both in cases of UAA and CAA, the role of drain placement after appendectomy, and the technique for closure of appendiceal stump. Finally, they underlined the potential beneficial results of endoscopic appendectomy for UAA, which seems to become the future intervention of AA [19].

## 5. Acute Appendicitis: Focus on the Parasite *Enterobius vermicularis*

Parasitic infection of the appendix is rare, with a reported incidence of 1.2% [20]. Among parasitic factors that may contribute to AA are *Schistosoma* spp., *Taenia* spp., *Ascaris lumbricoides*, and *Enterobious vermicularis* [20]. Parasites may cause obstruction of the appendiceal lumen without acute inflammation or by generating an inflammatory process [17]. However, whether a parasitic infestation may lead to AA or not is arguable.

Pogorelić et al. (contributor 4) investigated the frequency of *Enterobius vermicularis*, a parasite that may interfere with an unexpected appendectomy finding. The authors suggest a suspicion of the parasite in pediatric patients presented for AA with mild symptoms, a lower appendicitis inflammatory response score, a lower diameter of the appendix, and mild eosinophilia. 

## 6. Imaging Investigation: Focus on Clinical Observation 

Preoperative imaging is a key element compound in the diagnosis of AA, aiming to reduce unnecessary appendectomies. Ultrasound (U/S) and computed tomography (CT) are the most popular imaging options for the evolution of patients with suspected AA (1). However, according to a recent systematic review, no imaging tool can precisely omit complicated cases [21]. Luksaite-Lukste et al. (contributor 5), in a randomized controlled study, investigated the effectiveness of close observation with serial laboratory examination and U/S versus CT scan in adults suspected with AA. The authors found that close observation has similar sensitivity and specificity rates with repeated CT scan evaluation (97.7% and 96.4% vs. 96.7% and 95.8%), thus reducing the number of unnecessary CT scans without increasing the rates of negative appendectomies or the number of complications.

## 7. Acute Appendicitis: Focus on Hidden Appendicolith

Appendicoliths represent fecal calcified deposits in the lumen of the appendix [22]. Ranieri et al. in a cohort study, reported a prevalence of 38.7% (96/248) among adult patients diagnosed with AA [23]. There is evidence that the presence of an appendicolith in the lumen of the appendix decreases the failure of the conservative approach to AA, thus increasing the number of appendectomies [24]. However, in some cases, an incidental appendicolith in patients without AA has also been reported [25].

Dölling et al. (contributor 6), focused their investigation on the presence of hidden appendicoliths and their impact on the severity and treatment of AA. In their prospective study, incision of the appendix after appendectomy revealed a higher rate of appendicoliths, which could not be detected with conventional CT scans. Furthermore, the larger size of the appendicolith was a predictor factor of CAA. Their work provides new data on the prevalence of appendicoliths in AA. They suggest the use of more refined imaging modalities that could detect the hidden appendicoliths. 

## 8. Clinical Decision Rules in the Diagnosis of AA: Focus on Artificial Intelligence Information

Advances in Artificial Intelligence (AI) have begun to expand the traditional diagnostic tools of AA. The term concerns machine capacity to reproduce the human perceivability to perform projects autonomously in order to eliminate weaknesses in the diagnosis of AA [26]. AI enhances diagnostic accuracy by enrolling data from previous experience and producing new algorithms [27]. Two recent studies [28,29] support the introduction of AI in the diagnosis of AA, showing that AI surpasses traditional scoring systems and prevents unnecessary appendectomies and decreases the anxiety of patients and the financial burden of the health system. In the current issue, Dogan et al. (contributor 7) investigated the integration of a hybrid convolutional neural network model with ensemble learning techniques to improve the detection of AA from CT images. They found a success rate of 96% for the patient who performed a deep learning analysis of CT images vs. 83.3% with radiologically suspicious findings considered normal.

In conclusion, although AA has been studied for more than a century and the literature contains thousands of published articles, the diagnosis and treatment of AA continue to be challenging, and concerns still exist about the optimal management of AA. The studies added in this Special Issue help to narrow the gaps of our knowledge in the topic of AA and give the opportunity for further research on the topic.
